# Miliary Tuberculosis with Paraspinal Collection and Tuberculoma

**DOI:** 10.4269/ajtmh.23-0083

**Published:** 2023-07-03

**Authors:** Ashraf Ahmed, Saeed Mohammed, Mohamed Sadek

**Affiliations:** ^1^ Department of Internal Medicine, Hamad Medical Corporation, Doha, Qatar;; ^2^ Department of Diagnostic Radiology, Hamad Medical Corporation, Doha, Qatar

**Figure 1.**
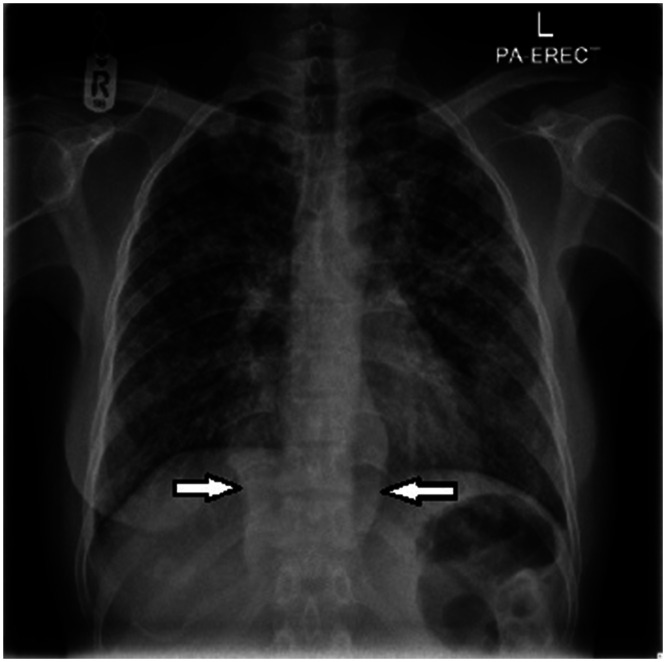
A chest X-ray showing extensive bilateral discrete and confluent nodular opacities in bilateral lungs. A bilateral paraspinal shadow is seen in the lower thoracic spine (arrows).

**Figure 2.**
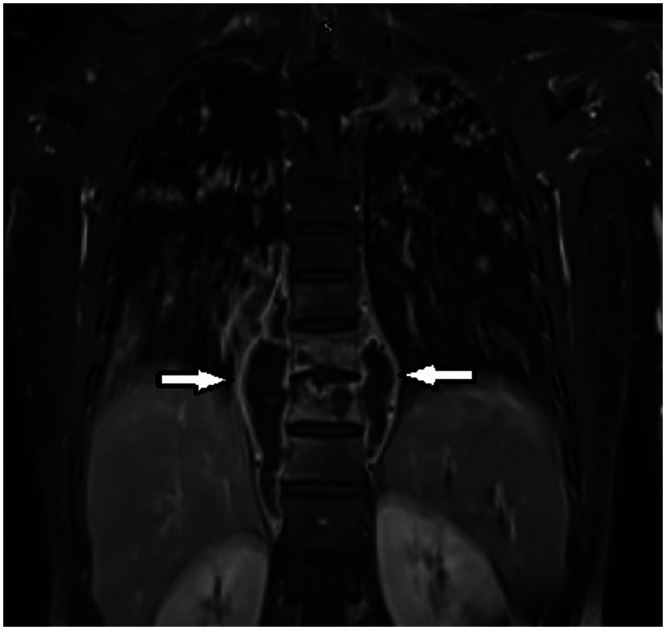
A spinal magnetic resonance imaging sagittal view showing heterogeneous enhancement of the T9, T10, and T11 vertebral body and bilateral paraspinal collection/cold abscess at the same level anteriorly (arrows).

**Figure 3.**
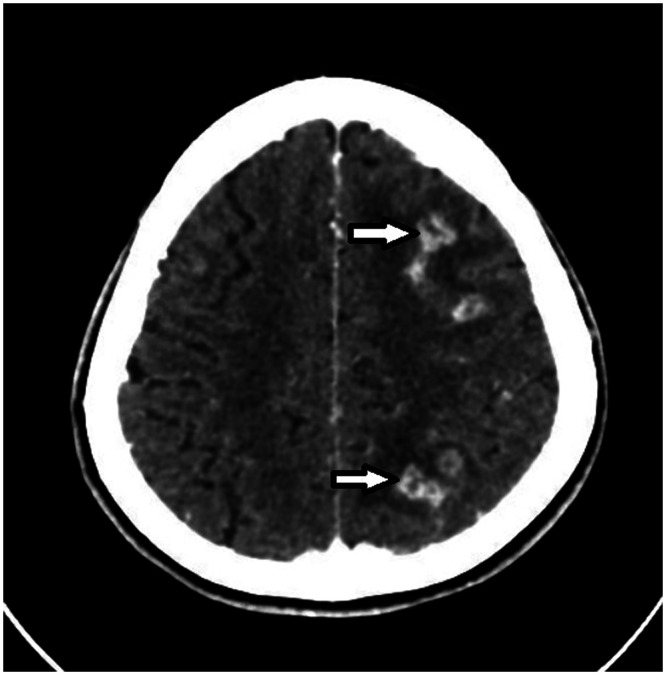
A computed tomography head scan with contrast showing multiple cortical/subcortical ring-enhancing lesions in the frontoparietal lobe with surrounding vasogenic edema (arrows).

A 35-year-old woman with no medical history presented complaining of fever, dry cough, and significant weight loss for 1 month, along with mild progressive shortness of breath. There was no hemoptysis or chest pain. The review of her systems was completely unremarkable. On examination, she was febrile, had a temperature of 38.1°C, heart rate of 109 beats per minute, and blood pressure of 103/67 mm Hg, and her oxygen saturation was 98% on room air. Chest examination showed diffuse inspiratory crackles bilaterally. Blood laboratory tests showed a normal leukocyte count, iron-deficiency anemia, and reactive thrombocytosis. The chest X-ray showed evidence of miliary tuberculosis (TB); however, a paraspinal homogeneous, hyperdense infiltrate was noted (Figure 1). A TB workup and a magnetic resonance imaging spine with contrast were done and showed paraspinal collection extending from T8 to L2 (Figure 2), with evidence of spondylodiscitis highly suggestive of TB. Meanwhile, the sputum acid-fast bacillus (AFB) smear result was positive, as was the TB polymerase chain reaction (PCR) result. She was started on rifampicin, isoniazid, pyrazinamide, and ethambutol. On day 9, the patient complained of headache, and examination showed nuchal rigidity. A computed tomography (CT) scan of the brain was done and showed evidence of tuberculoma (Figure 3), and hence dexamethasone was started. Five days later, the paraspinal collection was drained, guided by interventional radiology, and the AFB smear and PCR were reported to be positive from the collection. The patient continued on anti-TB medications; her headache and neck rigidity improved, and there was no back pain. The HIV test was negative and hemoglobin A1c was normal.

Miliary TB results in an extensive lymphohematogeneous spread of *Mycobacterium* TB, which seeds into different organs.^1^ Cold abscesses are commonly pyogenic and secondary to septic emboli from distant infections.^2^ Diagnosis is based on the local symptoms and imaging, mainly MRI, and management is typically CT-guided drainage and antimicrobial.^2^^,^^3^ A cold abscess might be missed in the absence of local symptoms, and any suspicious lesions on the X-ray should raise concerns for further evaluation and management.^4^
